# Pathological diagnosis experience and literature review of four cases suspected Lynch-like syndrome

**DOI:** 10.3389/fonc.2025.1608253

**Published:** 2025-11-06

**Authors:** Bo Cheng, Shan Liu, Shanshan Ding, Lanju Quan, Jinhong Liu, Lin Xu, Huan Zhao, Jing Guo, Suozhu Sun

**Affiliations:** Department of Pathology, People’s Liberation Army (PLA) Rocket Force Specialized Medical Center, Beijing, China

**Keywords:** Lynch-like syndrome, MMR germline mutation, MMR biallelic gene somatic mutation, histopathology, molecular pathology

## Abstract

**Background:**

Among CRC patients with mismatch repair protein deficiency or microsatellite instability (MSI), up to 50% of cases lack germline mutations in MMR genes, *BRAF* mutations, or *MLH1* promoter methylation. Such cases are defined as Lynch-like syndrome (LLS). LLS is a heterogeneous group of diseases that may include all the patients with cancers of the Lynch syndrome spectrum with MSI in which we don’t find a pathogenic variant in MMR genes. Although various methods have been proposed to distinguish Lynch and Lynch-like Syndrome, there is still a lack of consensus on the precise classification of these patients.

**Methods:**

Four cases of suspected Lynch-like syndrome encountered in daily clinical pathological diagnostic work were reported. The histopathological characteristics and molecular pathological changes of related tumors were analyzed, and the diagnosis and treatment progress of this disease were reviewed via literature.

**Results:**

Combined with clinical findings and molecular pathological tests, 2 cases were diagnosed as Lynch-like syndrome (LLS), and 2 case was diagnosed as Lynch syndrome with atypical phenotype. Lynch-like syndrome-related tumors can occur in the colorectum and extraintestinal organs. Colorectal tumors show no specific locational or histological features, while extraintestinal tumors often exhibit poor differentiation and abundant interstitial lymphocyte infiltration. Patients with Lynch-like syndrome all exhibit tumoral lesions with loss of MMR protein (MLH1, PMS2, MSH2, MSH6) expression, microsatellite instability (MSI-L/MSI-H), wild-type *BRAF*, and negative *MLH1* promoter methylation. However, heterogeneity exists in MMR protein expression, MSI status, and *MLH1* promoter methylation among tumors at different sites in the same patient. No germline pathogenic mutations in MMR genes were detected in any Lynch-like syndrome, but one cases showed variant of uncertain significance in MMR, and two case (Lynch syndrome with atypical phenotype) had likely pathogenic mutation in *MLH1.*

**Conclusion:**

Extraintestinal tumors associated with Lynch-like syndrome mostly exhibit histopathological characteristics and MMR/MSI changes similar to classic Lynch syndrome, but without pathogenic germline MMR mutations or *MLH1* promoter methylation. Some suspected Lynch-like syndromes with likely germline pathogenic MMR mutations may represent Lynch syndrome with atypical phenotype. Most cases lack germline MMR mutations in normal tissues but harbor somatic MMR mutations in tumor tissues. Germline or somatic mutations in other genes related to MMR function may be observed in some cases.

## 1 Introduction

Lynch syndrome (LS), caused by germline mutations in mismatch repair (MMR) genes, leads to DNA repair dysfunction and is characterized by familial aggregation and early onset. Accurate diagnosis is crucial for family screening and clinical intervention. However, some patients exhibit clinical features similar to LS (such as early-onset disease, multiple primary cancers, or family history) but lack detectable germline MMR mutations; these cases are termed “Lynch-like syndrome” (1). Currently, this field faces challenges: definitions remain inconsistent, diagnostic criteria lack consensus, and systematic research on pathological features is insufficient. Most existing studies are based on small samples, lacking comprehensive summaries of clinicopathological characteristics (2–4). Therefore, this study retrospectively analyzed the clinical data, pathological features, and immunohistochemical profiles of four cases of clinical suspected Lynch-like syndrome, combined with a literature review, to explore their clinicopathological characteristics and provide references for differential diagnosis and clinical management.

## 2 Materials and methods

### 2.1 Case collection

Four cases of suspected Lynch-like syndrome diagnosed in the Department of Pathology, PLA Rocket Force Specialized Medical Center, between January and October 2024 were enrolled. All patients had tumor lesions characterized by loss of MMR protein (MLH1, PMS2, MSH2, MSH6) expression, microsatellite instability (MSI-L/MSI-H), wild-type *BRAF*, and negative *MLH1* promoter methylation. Each case was re-evaluated and confirmed by two pathologists at or above the deputy chief physician level, and the study was approved by the institutional ethics committee with written informed consent obtained from all patients.

### 2.2 Immunohistochemical detection of mismatch repair proteins

Immunohistochemistry was performed using a Roche BenchMark XT automated staining system with the EnVision two-step method. Primary antibodies (MLH1, PMS2, MSH2, MSH6) and secondary antibodies were provided by Roche Biotechnology Development Co., Ltd. Interpretation criteria: Positive signals for MLH1, PMS2, MSH2, and MSH6 were nuclear. Tumor cells with positive staining showed tan-colored nuclei, while adjacent normal epithelial or stromal cells served as internal positive controls (tan nuclei). Negative tumor cells showed no nuclear staining, but normal cells in the vicinity exhibited tan nuclear staining.

### 2.3 *MLH1* promoter methylation analysis

DNA was extracted from tumor tissues, and its concentration and purity were measured using a UV spectrophotometer. Bisulfite modification and modified DNA purification were performed using the EZ DNA Methylation Gold™ Kit (ZYMO RESEARCH, USA) according to the manufacturer’s instructions. *MLH1* methylation was detected by quantitative real-time PCR on a 7500 ABI fluorescence instrument, with *COLO2A1* as an internal reference. Primer and probe sequences were as follows:

- Forward: 5’-CGTTATATATCGTTCGTAGTATTCGTGTTT-3’
- Reverse: 5’-CTATCGCCGCCTCATCGT-3’
- Probe: 5’-6FAM-CGCGACGTCAAACGCCACTACG-TAMRA-3’

Reaction system (20 μl):

5.2 μl ddH_2_O, 2 μl modified DNA, 10 μl Premix Ex Taq™ Hot Start, 1.2 μl forward primer, 1.2 μl reverse primer, 0.4 μl probe.

Thermal cycling conditions:

50°C for 2 min → 95°C for 10 min → (95°C for 15 s → 60°C for 1 min) × 40 cycles.

### 2.4 Germline and somatic mutation analysis of MMR genes

Peripheral blood samples and paraffin-embedded primary/metastatic tumor tissues were collected. Germline and somatic mutations in MMR genes were detected by next-generation sequencing (NGS) whole-exome sequencing, with concurrent evaluation of tumor MSI status. Detection kits were purchased from Shanghai Kunyuan Gene Technology Co., Ltd. and Beijing Novogene Co., Ltd., respectively. DNA extraction, library preparation, sequencing, and bioinformatics analysis were performed according to the manufacturers’ protocols. The gene list includes 22 genes associated with hereditary digestive system tumors, such as *APC, ATM, AXIN2, BLM, BMPR1A, CHEK2, EPCAM, GALNT12, GERM1, MLH1, MSH2, MSH3, MSH6, MUTYH, NTHL1, PMS2, POLD1, POLE, PTEN, SMAD, STK11, TP53.* In accordance with the ACMG/AMP Standards and Guidelines for the Interpretation of Sequence Variants, variants are ultimately classified into 5 categories (pathogenic, likely pathogenic, uncertain significance, likely benign, benign) through the “weighted evaluation” of “pathogenic evidence” and “benign evidence”.

### 2.5 Microsatellite instability detection in tumor tissues

MSI was assessed using the 2B3D fluorescence quantitative PCR-capillary electrophoresis method with markers BAT-25, BAT26, D2S123, D17S250, and D5S346 (kit from Shanghai Tongshu Biotechnology Co., Ltd.). MSI-H was defined as instability in ≥2 markers, MSI-L as instability in 1 marker, and MSS as stable in all markers.

## 3 Results

### 3.1 Clinical features

Case 1: A 53-year-old male presented two days after a rectal space-occupying lesion was detected on physical examination. Electronic colonoscopy revealed a circumferential mucosal elevation in the rectum, 13–16 cm from the anus, with luminal stenosis. He underwent laparoscopic anterior resection of rectal cancer, abdominal lymph node dissection, and lysis of intestinal adhesions.

Case 2: A 62-year-old female was admitted with postmenopausal intermittent vaginal bleeding for two years, worsening over the past four months. PET-CT showed increased FDG uptake in the uterine cavity and the descending colon, suspicious for malignancy.

Case 3: A 37-year-old female presented with menstrual irregularities for one year and intermenstrual discharge for two months. Pelvic ultrasound showed a heterogeneous echo at the anterior cervical lip (2.8×2.8×2.0 cm, ill-defined and irregular), confirmed as a 2.9×2.1 cm mass by pelvic MRI. Breast ultrasound revealed a left breast hypoechoic nodule (15×10×13 mm, BI-RADS 4a). She underwent comprehensive staging surgery for endometrial cancer and left breast conservative radical resection with sentinel lymph node biopsy.

Case 4: A 43-year-old female was admitted three days after multiple colonic polyps were detected on physical examination. Colonoscopy showed dozens of polypoid elevations (0.2–2.5 cm) 40–80 cm from the anus, with the largest lesion being 2.5×1.2 cm (pedunculated). Initial colonoscopic diagnosis was “multiple colonic polyps, nature pending,” with a clinical suspicion of familial adenomatous polyposis. She underwent laparoscopic total colectomy and ileorectal anastomosis.

### 3.2 Macroscopic and microscopic findings

Case 1: A circumferential ulcerative mass was present 3 cm from the distal resection margin, measuring 6×5×1 cm, with a necrotic, depressed center. Microscopically, tumor cells formed glandular-tubular structures, invading through the entire intestinal wall. The tumor stroma showed dense lymphocytic infiltrate, with tertiary lymphoid follicles observed at the invasion margin. Pathological diagnosis: “moderately differentiated tubular adenocarcinoma of the rectum.”

Case 2: Submitted specimens included a colonic segment, total uterus and bilateral adnexa.

- Colonic tumor: Glandular-tubular architecture with focal cribriform fusion, invading through the intestinal wall. Diagnosis: “moderately differentiated colonic adenocarcinoma.”
- Endometrial tumor: Solid nests invading myometrium, with intravascular tumor thrombi. Diagnosis: “poorly differentiated endometrioid adenocarcinoma of the endometrium.”

Case 3: Specimens included total hysterectomy, bilateral adnexa, and left breast tumor.

- Uterine tumor: Glandular-tubular and solid nests with focal keratin pearls, dense lymphocytic infiltrate between nests, invading uterine body and cervix. Diagnosis: “moderately to poorly differentiated endometrioid adenocarcinoma with focal adenosquamous features.”
- Breast tumor: Round/polygonal cells in solid nests, rich lymphocytic infiltrate. Diagnosis: “invasive breast carcinoma with medullary features.”

**Figure 1:**
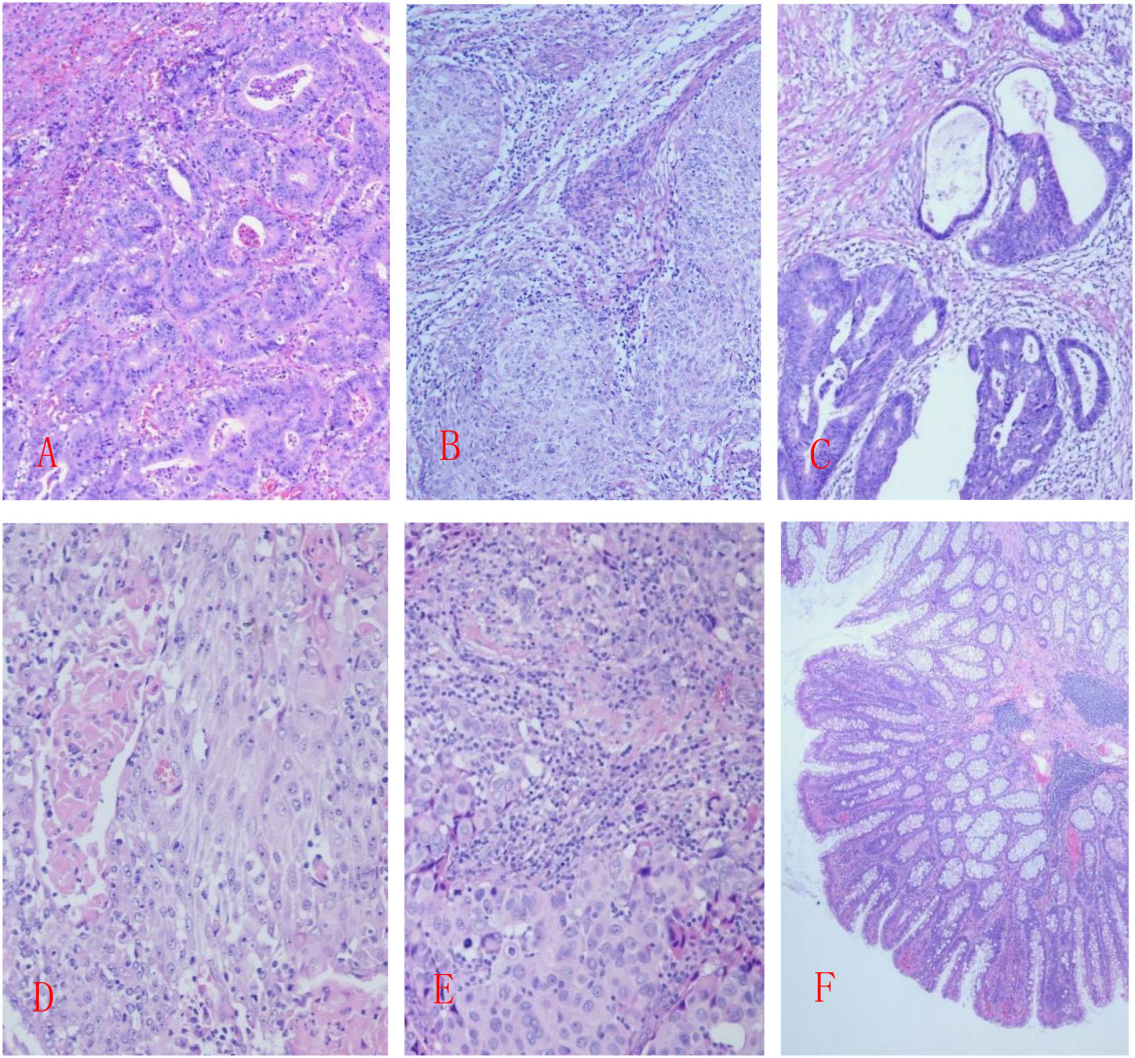
**(A)** Case 1: Moderately differentiated tubular adenocarcinoma of the rectum; **(B)** Case 2: Poorly differentiated endometrioid adenocarcinoma; **(C)** Case 2: Moderately differentiated adenocarcinoma of the colon; **(D)** Case 3: Moderately to poorly differentiated endometrioid adenocarcinoma, partially with adenosquamous carcinoma structure; **(E)** Case 3: Invasive carcinoma of the breast with medullary features; **(F)** Case 4: Low-grade adenomatous polyp of the colon; HE staining, 20X.

Case 4: The resected colon (55 cm long, 3–7 cm diameter) included a 6 cm appendix 5 cm from the proximal margin. Fifteen polyps (millet to rice grain size) were scattered, with normal remaining mucosa. Tumor cells showed glandular-tubular architecture with mild nuclear atypia. Diagnosis: “low-grade adenomatous polyp of the colon” (**Figure 1**).

### 3.3 Immunophenotype

Case 1: Tumor cells showed positive expression of CK20, CDX2, MLH1, and PMS2, with negative staining for MSH2 and MSH6.

Case 2:

- Endometrial tumor cells: Positive for CK7, ER, PR, MLH1, and PMS2; negative for CK5/6, P63, P40, PTEN, PAX2, MSH2, and MSH6.
- Colonic tumor cells: Positive for CK20, CDX2, MLH1, PMS2, MSH2, and MSH6; negative for CK7.

Case 3:

- Cervical tumor cells: Positive for CK7, CK5/6, P63, P40, ER, PR, MSH2, and MSH6; negative for PTEN, PAX2, MLH1, and PMS2.
- Breast tumor cells: Positive for CK7, E-Cadherin, GATA3, ER, PR, HER2, MSH2, and MSH6; negative for CK5/6, P63, P40, MLH1, and PMS2.

**Figure 2:**
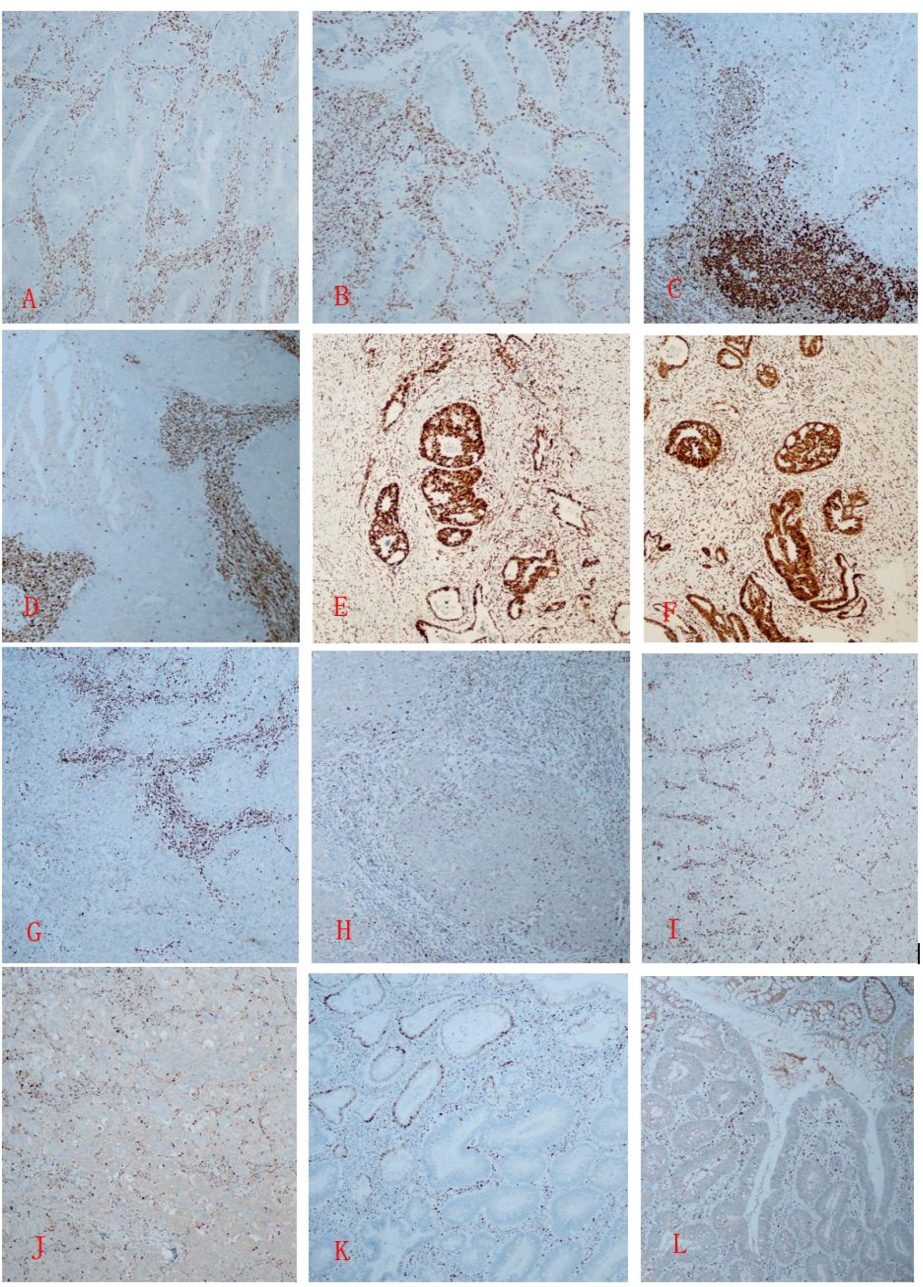
**(A, B)** In Case 1, immunohistochemistry of the rectal cancer tissue shows MSH2 **(-)** and MSH6 (-); **(C, D)** In Case 2, immunohistochemistry of the poorly differentiated endometrioid adenocarcinoma tissue shows MSH2 (-) and MSH6 (-); **(E, F)** In Case 2, the colon cancer cells show MSH2 (+) and MSH6 (+); G, **(H)** In Case 3, the cervical tumor tissue shows MLH1 (-) and PMS2 (-); **(I, J)** In Case 3, the breast tumor tissue shows MLH1 (-) and PMS2 (-); **(K, L)** In Case 4, The colonic adenomatous polyp shows MLH1 (-) and PMS2 (-). Immunohistochemistry, one-step method, X20.

Case 4: Tumor cells were positive for CK20, CDX2, MSH2, and MSH6, with negative expression of MLH1 and PMS2. (**Figure 2**)

### 3.4 Molecular pathological characteristics

Case 1: Germline mutation analysis of peripheral blood revealed a variant of uncertain significance (VUS) in *PMS2*. NGS sequencing of rectal cancer tissue detected a somatic pathogenic mutation in *MSH2, MSH6* and variant of uncertain significance (VUS) in *PMS2*. The tumor was microsatellite instability-high (MSI-H), with no *BRAF* mutation or *MLH1* promoter methylation.

Case 2: No germline mutations in MMR genes were detected in peripheral blood. NGS of endometrial cancer tissue identified a pathogenic mutation in *POLE*, while colon cancer tissue showed a pathogenic mutation in *TP53*; no MMR gene alterations were found. Endometrial cancer was microsatellite instability-low (MSI-L), and colon cancer was microsatellite stable (MSS).

Case 3: Variant of likely pathogenic mutation in *MLH1* were detected in peripheral blood, uterine, and breast tumor tissues. Tumor tissue exhibited MSI-H, with no *BRAF* mutation or *MLH1* promoter methylation.

Case 4: Germline analysis of peripheral blood showed likely pathogenic mutation in *MLH1*. NGS of colonic tumor tissue revealed a pathogenic mutation in *TP53* and a likely pathogenic mutation in *MLH1*. The tumor was MSI-H, with no *BRAF* mutation or *MLH1* promoter methylation. (**Tables 1**, **2**)

**Table 1:** Molecular pathological test results of cases 1, 2, 3 and 4.

Case	Genetic changeTumor location	MSI	*BRAF*	*MLH1* methylation	Tumor somatic mutations	MMR Germline mutations
Case1	Rectum	MSI-H	–	–	*MSH2* pathogenic mutation *MSH6* pathogenic mutation *PMS2* variant of uncertain significance	*PMS2* variant of uncertain significance
Case2	Endometrium	MSI-L	–	–	*POLE* pathogenic mutation	_
	Colon	MSS	–	–	*P53* pathogenic mutation	
Case3	Endometrium	MSI-H	–	–	*MLH1* likely pathogenic mutation	*MLH1* likely pathogenic mutation
	Breast	MSI-H	–	–	*MLH1* likely pathogenic mutation	
Case4	Colon	MSI-H	–	–	*P53* pathogenic mutation *MLH1* likely pathogenic mutation	*MLH1* likely pathogenic mutation

**Table 2:** List of somatic and germline gene mutations in cases 1, 2, 3, and 4.

Case	Genetic changeTumor location	Gene	Location	Exon	Mutation frequency	HGVSc	HGVSp	ACMG/AMP
Case1	Rectum	somatic mutations	*MSH2*	Chr2:47656951	7	20.94%	c.1147C>T	p.Arg383Ter	Pathogenic
			*MSH6*	Chr2:48030639	5	37.00%	c.3261delC	p.Phe1088serfsTer2
			*PMS2*	Chr7:48030639	6	45.03%	c.598G>A	p.Val200Ile	Variant of Unknown Significance
	germline mutations		*PMS2*	Chr7:48030639	6	47.33%	c.598G>A	p.Val200Ile	Variant of Unknown Significance
Case2	Endom-etrium	somatic mutations	*POLE*	Chr12:133250250	13	6.81%_	c.1270C>G	p.Leu424Val	Pathogenic
	Colon	somatic mutations	*P53*	Chr17:7578449	5	5.06%	c.481G>A	p.Ala161Thr	Pathogenic
	germline mutations		–						
Case3	Endom-etrium	somatic mutations	*MLH1*	Chr3:37081733	14	51.91%	c.1615G>C	p.Ala539Pro	Likely Pathogenic
	Breast	somatic mutations	*MLH1*	Chr3:37081733	14	52.35%	c.1615G>C	p.Ala539Pro	Likely Pathogenic
	germline mutations		*MLH1*	Chr3:37081733	14	50.14%	c.1615G>C	p.Ala539Pro	Likely Pathogenic
Case4	Colon	Somaticmutations	*MLH1*	Chr3:37038149	2	51.23%	c.161-164dupGAGG	p.Leu56ArgfsTer24	Likely Pathogenic
	*P53*	Chr17:7565337	10	32.00%	c.1024C>T	p.Arg342Ter	Pathogenic
	germline mutations		*MLH1*	Chr3:37038149	2	49.59%	c.161-164dupGAGG	p.Leu56ArgfsTer24	Likely Pathogenic

## 4 Discussion

Lynch-like syndrome (LLS) refers to a clinical phenotype with characteristics similar to Lynch syndrome, but without detectable germline pathogenic/likely pathogenic mutations in mismatch repair genes (MMR). LLS represents a heterogeneous group of disorders. Although various approaches have been proposed to differentiate between hereditary and sporadic cases of LLS, there remains a lack of consensus on the precise classification of such patients. Regarding the definition of Lynch-like syndrome (LLS), some scholars argue that it should refer to cases who have a suspect of hereditary cancer (such as early-onset disease, multiple primary cancers, or a family history of the disease) but in which we cannot find a pathogenic variant in MMR genes. In this definition, the presence of a cancer with MSI is not sufficient to classify the patients as LLS, but they need to meet Amsterdam and/or Bethesda criteria (1). The other definition, followed by some authors as Picó et al. include all the patients who present with tumor microsatellite instability (MSI) or loss-of-expression MMR proteins but without evidence of germline pathogenic mutation in MMR genes. This is a broader definition of LLS because all the patients with colorectal/endometrial cancer with MSI without MMR gene mutations are included. it is important to adequately address the existence of variants of unknown significance (VUS) following international guidelines for classification. Inadequately classified VUS in MMR genes could also be a cause of LLS (5). This article is based on the expanded definition of Lynch-like syndrome (LLS), four clinical cases pathologically suspected Lynch-like syndrome were selected to analyze the histopathological features and molecular pathological alterations of the associated tumors. Combined with clinical findings and molecular pathological tests, 2 cases were diagnosed as Lynch-like syndrome (LLS), and 2 cases was diagnosed as Lynch syndrome with atypical phenotype.

In case 1, both gross and microscopic findings showed a classic moderately differentiated tubular adenocarcinoma of the rectum. However, IHC testing revealed the relatively rare loss of MSH2/MSH6 expression. Microsatellite instability (MSI) testing confirmed the tumor to be microsatellite instability-high (MSI-H). Tests for *MLH1* promoter methylation and *BRAF* gene mutation did not support a diagnosis of classic sporadic CRCs with dMMR. Since only a variant of uncertain significance (VUS) in *PMS2* was detected in the MMR germline mutation analysis, Lynch syndrome was excluded. Combined with the detection of somatic pathogenic mutations in *MSH2* and *MSH6* in tumor tissue, the case was diagnosed as Lynch-like syndrome. In clinical practice, classic sporadic MSI-H colorectal cancers are predominantly caused by *MLH1* promoter methylation and often accompanied by *BRA*F gene mutations (6). Lynch syndrome caused by germline pathogenic mutations in *MSH2* or *MSH6* typically presents with synchronous or metachronous extraintestinal malignancies and occurs at a older age (7). Although a germline VUS in *PMS2* was detected in this case, its clinical significance remains unclear and cannot serve as a basis for diagnosing Lynch syndrome.

In case 2, the patient presented with synchronous poorly differentiated endometrioid adenocarcinoma and moderately differentiated tubular adenocarcinoma of the colon. The endometrial tumor tissue showed loss of MSH2/MSH6 protein expression and microsatellite instability-low (MSI-L). Despite the relatively older age at tumor onset, clinical and pathological features highly suggested Lynch syndrome. Further evaluation revealed that the colon cancer exhibited preserved MSH2/MSH6 expression and was microsatellite stable (MSS), lacking the molecular pathological features of Lynch syndrome-related tumors. Germline mutation analysis of MMR genes in peripheral blood was negative, excluding Lynch syndrome. NGS sequencing of tumor tissue detected pathogenic mutations in *POLE* gene in the endometrial cancer, and a *P53* pathogenic mutation in the colon cancer. Given that *POLE* gene mutations can induce loss of MMR protein expression, this case was diagnosed as Lynch-like syndrome attributed to a *POLE* pathogenic mutation. Colorectal and endometrial cancers are the most common Lynch-related tumors. Although the patient had synchronous endometrial and colon cancers, the MMR protein expression and microsatellite status differed significantly between the two tumors, and No germline mismatch repair (MMR) gene mutations were detected, which is inconsistent with the molecular characteristics of Lynch syndrome. Somatic mutations in *POLE* are frequently observed in endometrial cancer. Studies have reported that *POLE* mutations can lead to loss of MMR protein expression and microsatellite instability (8), consistent with the findings in this case. Poorly differentiated endometrioid adenocarcinoma is relatively rare; tumors with MMR protein loss often exhibit phenotypes of poor differentiation and medullary carcinoma, with rich lymphocytic stroma, and have a better prognosis than conventional poorly differentiated adenocarcinomas (9).

Case 3 involved a young female with synchronous endometrioid adenocarcinoma (with focal adenosquamous differentiation) and invasive ductal carcinoma of the breast. Both tumors exhibited loss of MLH1/PMS2 protein expression and microsatellite instability-high (MSI-H), with no detectable *BRAF* mutations or *MLH1* promoter methylation. A likely pathogenic variant in the *MLH1* gene was identified via peripheral blood NGS sequencing, leading to an diagnosis of Lynch syndrome with atypical phenotype. Studies indicate that some hereditary cases may represent classic Lynch syndrome caused by rare MMR mutations (10). Although the patient had not developed colorectal cancer at the time of diagnosis, her father succumbed to gastric, colorectal, and bladder cancers before age 50, raising suspicion for Lynch syndrome due to unusual MMR mutations that cannot be definitively excluded. A comprehensive assessment should be conducted by integrating genetic function, variant characteristics, clinical data, and other relevant information to rule out Lynch syndrome with atypical phenotypes.Breast and endometrial cancers are common genetically associated malignancies. The former is primarily linked to hereditary breast and ovarian cancer syndrome (HBOC) caused by *BRCA1/2* germline mutations (11), while the latter is strongly associated with Lynch syndrome due to MMR gene mutations. Breast cancer is an infrequent extraintestinal manifestation of LS (12). In this case, both endometrial and breast tumors demonstrated loss of MLH1/PMS2 expression, MSI-H, and prominent lymphocytic infiltration in poorly differentiated regions—histological features consistent with Lynch-related tumors. This case was once misdiagnosed as Lynch-like syndrome due to the false reporting of peripheral blood MMR germline mutation test results as variants of unknown significance (VUS). Later, the diagnosis was corrected under the guidance of reviewing experts. This further confirms that extreme caution should be exercised in diagnosing Lynch-like syndrome for some cases with a clear family history and concurrent MMR germline mutations.

In case 4, multiple colonic polyps were identified during a physical examination, prompting a clinical diagnosis of “familial adenomatous polyposis (FAP)”. Microscopic examination revealed glands with low-grade dysplasia, pathologically diagnosed as “low-grade adenomatous polyps”. Tumor tissue exhibited loss of MLH1/PMS2 protein expression and microsatellite instability-high (MSI-H), with no detectable *BRAF* mutations or *MLH1* promoter methylation. Likely pathogenic variant in the *MLH1* gene was detected via germline mutation analysis of peripheral blood, No germline pathogenic mutations in FAP/MAP related genes such as *APC* , *MUTYH* and *NTHL1* were detected. The patient’s father has a clinical diagnosis of Familial Adenomatous Polyposis (FAP), but germline mutation testing for genes associated with hereditary digestive system tumors has not yet been performed. Hence, this case was pathologically diagnosed as Lynch syndrome with atypical phenotype. FAP is a rare autosomal dominant disorder characterized by numerous adenomatous polyps, predisposing to early-onset colorectal cancer (CRC). Approximately 70% of FAP patients exhibit extraintestinal manifestations, such as Gardner syndrome (13), Turcot syndrome (14), or gastric adenocarcinoma and proximal gastric polyposis (GAPPS) (15), all associated with germline mutations in the adenomatous polyposis coli (APC) gene. Mutations in *APC* are the primary cause of classic FAP (cFAP). However, cases lacking detectable *APC* mutations, termed APC(-)/cFAP, often harbor germline mutations in susceptibility genes, including *MUTYH* and *NTHL1*. Biallelic DNA mismatch repair (MMR) mutations can cause autosomal recessive APC(-)/cFAP, while autosomal dominant forms may arise from mutations in *POLE/POLD1*, *AXIN2*, or *DUOX2* (16). Although this case was finally diagnosed as Lynch syndrome with an atypical phenotype, it has reference significance for the differential diagnosis of Lynch-like syndrome. For cases with the same loss of mismatch repair protein expression and microsatellite instability phenotype, if a variant of unknown significance (VUS) in MMR genes is detected, the diagnosis should be Lynch-like syndrome.

Studies indicate that the LLS cohort and their first-degree relatives have a lower risk of CRC and other Lynch syndrome (LS)-related cancers compared to LS patients. Nevertheless, LLS patients face a higher CRC risk than sporadic cases (1, 17). María Dolores Picó et al. analyzed 160 LLS patients, reporting a mean age of onset of 55 years for LLS-related CRC, with 41% being female. Eleven percent met Amsterdam I/II criteria for LS, and 65% fulfilled revised Bethesda guidelines. Among LLS patients, 24% were identified during CRC screening. No significant differences were observed in gender, colonoscopy indications, immunohistochemistry results, pathological features, or personal history of CRC/other LS-related tumors between patients meeting Amsterdam/Bethesda criteria and those without a family history of CRC (5). Erell Guillerm et al. found six patients with double somatic hits, including one patient with mosaicism of a *de novo* pathogenic variant in MSH2 using tumoral NGS analysis of 16 patients with Lynch-like syndrome. This variant was transmitted to the patient’s offspring, which has significant implications for genetic counseling (18). Francesca Piriniet al investigate the causal mechanism of LLS by a comprehensive genetic and epigenetic approach.Their multigene panel analysis revealed the presence of pathogenic variants in non-mismatch repair (MMR) genes possibly predisposing to LLS. Their epigenetic analysis showed epivariations targeting genes associated with LS or DNA repair, most of them associated with the Fanconi Anemia pathway (19).

The etiology of LLS remains unclear, with four proposed mechanisms: First, the presence of alterations on MMR genes still considered as variants of uncertain significance (VUS) (18); second, some LLS patients may indeed be undiagnosed LS cases, as current technologies struggle to identify complex or recessive mutations. Structural variations (e.g., intronic regions, inversions, copy number variations [CNVs]) are rarely analyzed in routine genetic testing yet may underlie mutations in these patients (10, 20); Third, alterations in non-MMR genes like MUTYH, EXO1, POLE, POLD1, MCM, WRN, BARD1, RCF1, RPA1, MLH3, PPARG, CTC1, DCC, ALPK, PRKDC (19). Germline mutations in *MUTYH* and *POLE* have been reported in dMMR patients (21, 22); Fourth, additional mechanisms, such as the presence of constitutional epigenetic alterations, can cause a MMR-deficient phenotype. In sporadic cancers without MMR gene alterations but with loss of MMR protein expression, alternative molecular mechanisms (e.g., somatic oncogene alterations or epigenetic events outside the MMR system) may drive dMMR/MSI phenotypes. Such tumors, potentially of sporadic origin, might be excluded from LLS classification. However, this approach is problematic due to the lack of standardized protocols for distinguishing these cases. Classifying patients as sporadic or hereditary requires integrating clinical practice, comprehensive family pedigree analysis, and long-term follow-up to assess CRC/LS-related cancer incidence disparities (23–25).

This article reports four cases of suspected Lynch-like syndrome (LLS). Although the sample size is small, these cases are rare and represent the clinical heterogeneity and mechanistic complexity of LLS. Two cases are caused respectively by somatic mutations in the mismatch repair (MMR) system or *POLE* gene in tumor cells without clear family history of hereditary disease. The other two cases had a family history of tumors, but no clinical samples were obtained for systematic germline mutation testing in family members. Case 3 and 4 was diagnosed with Lynch syndrome with an atypical phenotype due to the detection of a likely pathogenic variant. These findings highlight that the diagnosis of Lynch-related syndromes (including Lynch-like syndrome and Lynch syndrome with atypical phenotypes) relies heavily on germline mutation testing results, and the lack of family member samples or differences in the nature of detected variants can directly affect the accuracy of diagnostic classification.

## Data Availability

The datasets presented in this study can be found in online repositories. The names of the repository/repositories and accession number(s) can be found in the article/supplementary material.
